# Choroidal Vascularity Index in CHM Carriers

**DOI:** 10.3389/fopht.2021.755058

**Published:** 2021-10-20

**Authors:** Dario Pasquale Mucciolo, Dario Giorgio, Myrta Lippera, Ilaria Passerini, Elisabetta Pelo, Francesca Cipollini, Andrea Sodi, Gianni Virgili, Fabrizio Giansanti, Vittoria Murro

**Affiliations:** ^1^ Department of Neuroscience, Psychology, Drug Research and Child Health, University of Florence, Florence, Italy; ^2^ Ophthalmology Unit, San Jacopo Hospital, Pistoia, Italy; ^3^ Department of Genetic Diagnosis, Careggi University Hospital, Florence, Italy; ^4^ Fondazione GB Bietti, Rome, Italy

**Keywords:** CHM, carriers, choroideremia, CVI, OCT, choroid

## Abstract

**Purpose:**

To assess the choroidal structure using the Choroidal Vascularity Index (CVI) and analyse choroidal changes in choroideremia (CHM) carriers.

**Material and Methods:**

Female CHM carriers, genetically characterized, and a control group were recruited at the Eye Clinic of Careggi Teaching Hospital, Florence. The patients underwent a complete ophthalmic evaluation and retinal imaging. In particular, the Stromal Area (SA), Luminal Area (LA), Total Choroidal Area (TCA), CVI, and Subfoveal Choroidal Thickness (SFCT) were calculated for each eye using Optical Coherence Tomography (OCT) examinations.

**Results:**

Twelve eyes of 6 CHM carriers and 14 eyes of 7 age-matched controls were analysed. The mean SFCT was 270.9 ± 54.3μm in carriers and 281.4 ± 36.8μm in controls (p = 0.564); LA was 0.99 ± 0.25mm^2^ and 1.01 ± 0.13mm^2^ (p = 0.172); SA was 0.53 ± 0.09mm^2^ and 0.59 ± 0.07mm^2^ (p = 0.075), and TCA was 1.53 ± 0.34mm^2^ and 1.69 ± 0.19mm^2^ respectively (p = 0.146). Mean CVI measured 64.03 ± 3.98% in the CHM carriers and 65.25 ± 2.55% in the controls (p = 0.360).

**Conclusions:**

The CVI and CVI-related parameters (SA, LA, and TCA) do not differ between CHM female carriers and controls. These findings reveal a preserved choroidal vasculature in eyes with RPE impairment and support the primary role of RPE in the pathogenesis of CHM disease.

## Introduction

Choroideremia (CHM) is an X-linked dystrophy characterized by a progressive and widespread degeneration of the Retinal Pigment Epithelium (RPE), Photoreceptors (PHR), and Choriocapillaris (CC). Its prevalence is estimated 1 in 50000–100000 (1). The disease is caused by a variety of mutations involving the CHM gene, located on chromosome Xq21.2 (1). The CHM gene encodes for Rab escort protein-1 (REP-1), involved in intracellular vescicular trafficking (2). Men are predominantly affected, while women are mainly asymptomatic carriers (1). The first symptom of choroideremia is development of nyctalopia during childhood, while peripheral vision loss frequently develops in the second decade of life (2). Good visual acuity is normally maintained until the fifth to seventh decade, when a rapid deterioration in central vision occurs, preceded by color vision deterioration (2). In CHM carriers, visual impairment is rarely present and it is typically associated with skewed X-inactivation (3). In fact, carriers usually have no symptoms or only mild to moderate night blindness. The earliest appearance of CHM on fundus examination is RPE pigment clumping (3). Successively, peripheral areas of atrophy develop. Although the atrophy progresses centripetally, an island of foveal tissue frequently persists. However, in advanced disease stages, it can reach the fovea. Differently from retinitis pigmentosa, in CHM larger choroidal blood vessels, retinal vessels, and optic disc normal configuration are preserved. Female carriers of CHM can express different degrees of chorioretinopathy, like areas of chorioretinal degeneration, yellowish drusen-like deposits, and peripheral pigmentary granularities (3). The diagnosis of CHM is based on typical fundus appearance and family history and confirmed by genetic testing. CHM has raised new interest since it is a promising candidate for successful gene therapy. Recently, the study of the choroid has been improved thanks to the Choroidal Vascularity Index (CVI), which has already been used for various retinal diseases (4–6). The CVI is defined as the proportion of the Luminal Area (LA) to the Total Choroidal Area (TCA), and therefore it studies quantitatively choroidal vascularity (4). In this study, we firstly have assessed the choroidal structure using the CVI and analysed choroidal changes in CHM carriers.

## Materials and Methods

### Study Population

Six female CHM carriers were recruited at the Regional Reference Center for Hereditary Retinal Degenerations at Careggi Teaching Hospital, Florence, Italy. Seven healthy female subjects of matching age and ethnicity were enrolled as a control group. The genetic examination was carried out at the Department of Genetic Diagnosis at Careggi Teaching Hospital. Accurate pharmacological and family histories were collected for each patient. All the patients underwent a complete ophthalmic examination, including Best Corrected Visual Acuity (BCVA), measurement of intraocular pressure, biomicroscopy of the anterior segment, and funduscopic examination. Moreover, all the patients underwent Fundus Autofluorescence (FAF) and color fundus photographs (ultra-wide-field digital scanning laser technology, Daytona, Optos), Swept Source Optical Coherence Tomography (SS-OCT), and OCT Angiography (Triton, Topcon Medical Systems Inc, Oakland, NJ, USA), Full-Field standard ERG according to ISCEV protocols (RETIMAX, Roland Consult, Brandenburg, Germany), Goldmann Visual Field. According to a classification used in previous studies (3, 7), four different patterns (A, B, C, D) describe the different appearances of the posterior pole in terms of RPE abnormalities for CHM carriers.

### Choroidal Thickness Assessment and CVI Calculation

**Figure 1:**
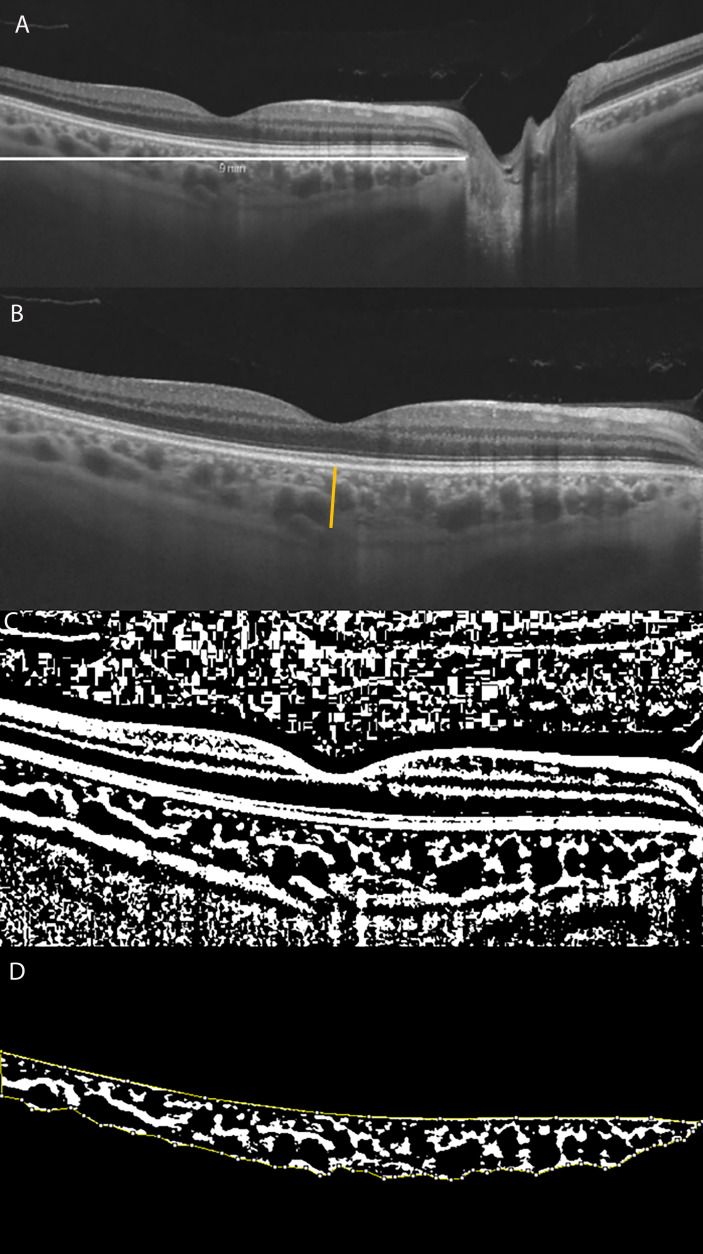
**(A)** Long horizontal OCT B-scan at the fovea of a control. **(B)** 9 mm Horizontal OCT B-scan at the fovea. The orange line indicates the SFCT. **(C)** The image was binarized using Niblack’s auto-local threshold to calculate the CVI; the binarization was performed using public domain software “Image J” through an automated function. The dark pixels represent the luminal area and the white pixels the stromal area. (**D**) After uploading the images on Image J, a polygon tool was used to select the total choroid area (TCA-area between the yellow lines), with the RPE representing the anterior boundary of the TCA and the scleral-choroidal interface as the posterior boundary of the TCA, across the entire length of the scan.

**Figure 2:**
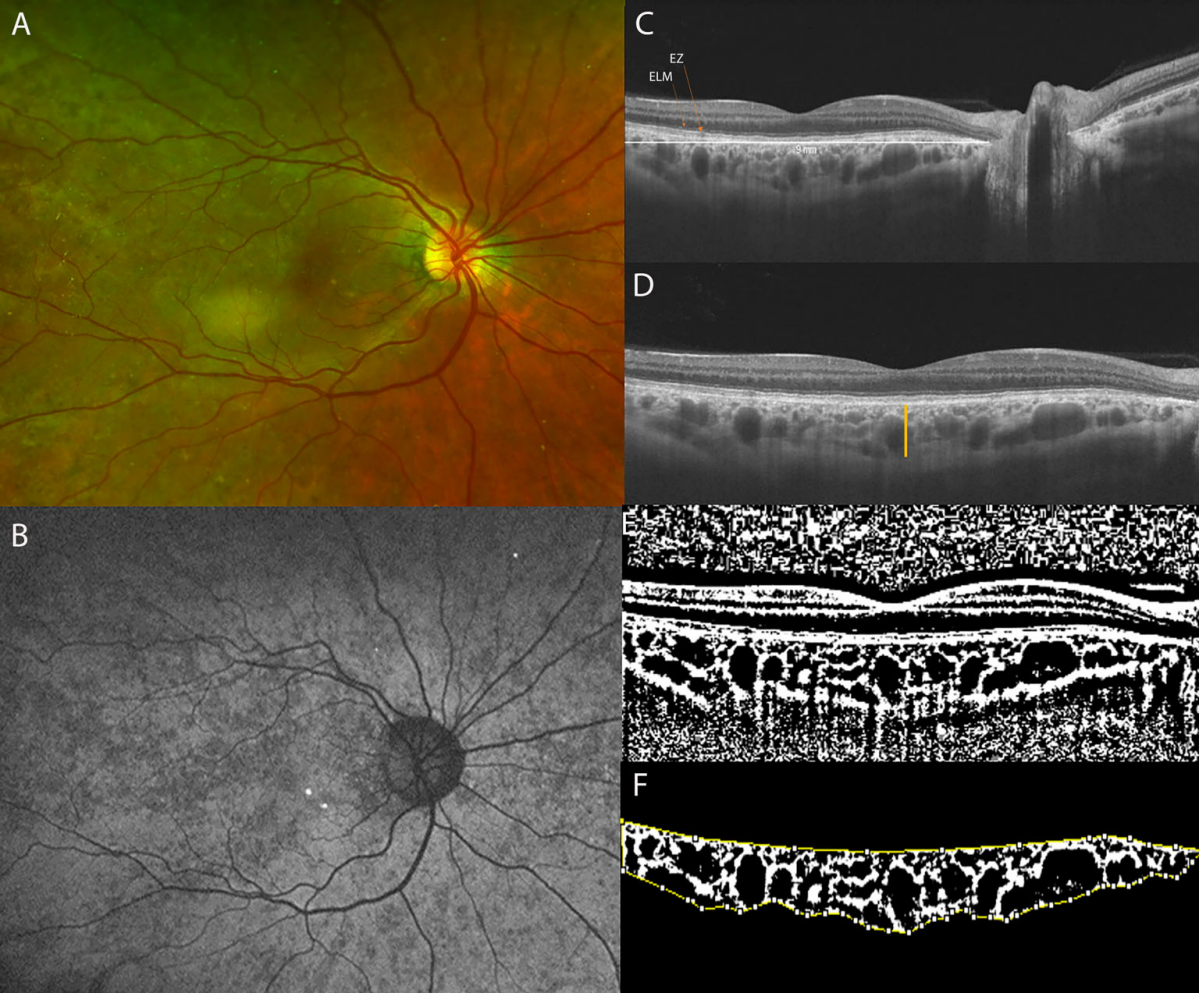
**(A)** Ultra-Widefield (UWF) color fundus imaging and **(B)** Fundus Autofluorescence (FAF) of the right eye of a 42-year-old CHM female carrier. UWF imaging shows large and irregular hypo-pigmented areas with yellowish deposits at the macula. **(C)** Long horizontal OCT B-scan at the fovea showing a preserved External Limiting Membrane (ELM) and slight irregularities of the Ellipsoid Zone (EZ) and RPE-Bruch’s membrane complex. **(D)** 9 mm horizontal OCT scan. The orange line indicates the SFCT. **(E)** The image was binarized using Niblack’s auto-local threshold to calculate the CVI; the binarization was performed using public domain software “Image J” trough an automated function. The dark pixels represent the luminal area and the white pixels the stromal area. **(F)** The CVI was calculated as the ratio between the Luminal Area (LA) and the total choroidal area (TCA). After uploading the images on Image J, a polygon tool was used to select the total choroid area (TCA-area between the yellow lines), with the RPE representing the anterior boundary of the TCA and the scleral-choroidal interface as the posterior boundary of the TCA, across the entire length of the scan.

**Figure 3:**
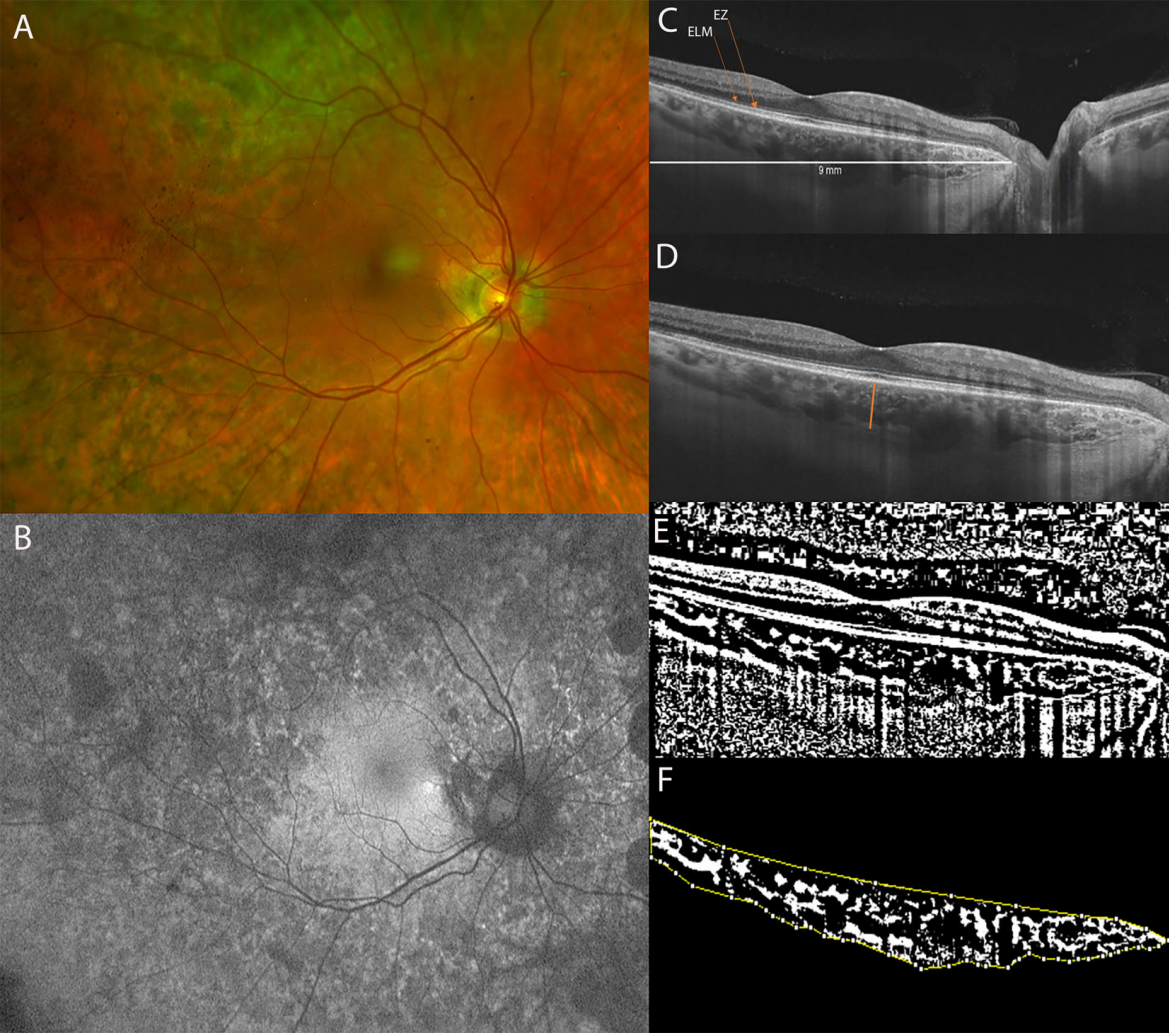
**(A)** Ultra-Widefield (UWF) color fundus imaging and **(B)** Fundus Autofluorescence (FAF) of the right eye of a 51-year-old female CHM carrier. FAF shows a normal appearance of the macula with multiple hypo-autofluorescent areas at the posterior pole and at the mid-periphery. **(C)** Long horizontal B-scan OCT passing through the fovea showing atrophy of the outer retinal layers (interdigitation zone, ellipsoid zone, outer nuclear layer) and RPE layer at the peripapillary area. **(D)** 9 mm horizontal B-scan OCT. The orange line indicates the SFCT. **(E)** The image was binarized using Niblack’s auto-local threshold in order to calculate the CVI. The binarization was performed using public domain software “Image J” through an automated function. The dark pixels represent the luminal area and the white pixels the stromal area. **(F)** The CVI was calculated as the ratio between the luminal area (LA) and the total choroidal area (TCA). After uploading the images on Image J, a polygon tool was used to select the total choroid area (TCA-area between the yellow lines), with the RPE as the anterior boundary of the TCA and the scleral-choroidal interface as the posterior boundary of the TCA, across the entire length of the scan.

High-resolution choroidal imaging was obtained acquiring a 12x9 mm-line OCT scan centered at the fovea for each eye (Triton, Topcon Medical Systems Inc, Oakland, NJ, USA) (**Figures 1**, **2**). Using the caliper tool present in the OCT software, the Subfoveal Choroidal Thickness (SFCT) was measured, at the level of the fovea, from Bruch’s Membrane to the sclero-choroidal interface. The subfoveal choroidal area with a width of 9 mm from the optic disc was considered the area of interest for measurement acquisitions (using line tool of software “Image J”). The binarization was performed using public domain software “Image J” (https://imagej.net/Welcome), as described by Agrawal et al. (8). Once the OCT scan was uploaded on “Image J” for each eye, the Total Choroidal Area (TCA) was measured across the entire length of the scan, using the RPE as anterior boundary and the scleral-choroidal interface as the posterior boundary. Successively, the image was converted into an 8-bit pixel resolution, and the mean pixel value was obtained for each point using Niblack auto local thresholding. Finally, the TCA was analysed with a Color Threshold Tool. The summary of the dark pixels constituted the Luminal Area (LA), while the summary of the light pixels represented the stromal area (SA); the ratio LA/TCA then revealed the CVI (**Figures 1**–**3**). Furthermore, the subfoveal choroidal area within a 3 mm diameter centered over the fovea (1.5 mm on either side of the fovea) was also selected and analysed according to the same method in CHM carriers to compare the choroidal OCT parameters between CHM carriers with and without central RPE abnormalities.

The results of the study are expressed as mean ± standard deviation (SD) in the case of quantitative variables. A linear mixed model was used for the statistical analysis and to compute p-values. Mixed models account for correlated data between eyes of the same individual, thus making more conservative assumptions than standard t-test ignoring correlation. Moreover, no normality assumption of the dependent variable is required, but only that the variances of the residuals don’t differ across covariate levels. This assumption, i.e. homoscedasticity of residuals, was assessed with graphical inspection, and finding residuals were similar for CHM and controls.

Ethical approval was waived by the local Ethics Committee of the University of Florence. The study adhered to the principles of the Helsinki Declaration and all the participants signed a written informed consent.

## Results

### Study Population

The study evaluates 12 eyes of 6 CHM carriers and 14 eyes of 7 age-matched controls. The mean age was 45.5 ± 16.3 years (range, 15–61 years) for CHM carriers and 45.6 ± 12.8 years (range, 18–54 years) for controls. All CHM carriers were genetically characterized (**Table 1**). The BCVA was 20/20 for all the eyes included in the study. All the patients were asymptomatic. The mean spherical equivalent was 0.08 ± 0.34 D (range 0.75; -0.25) for the CHM female carrier group and −0.71 ± 1.39 D (range 1, −2.75) for controls. The Goldmann visual field was normal in all patients of both groups. The anterior segment was unremarkable in the 2 groups (in the CHM carrier group 5 patients were phakic, with a transparent lens, while only 1 patient was pseudophakic). Fundoscopic examination did not reveal alterations in the controls. On the contrary, all the CHM carriers showed variable pigmentary alterations at the mid and far peripheral retina. In particular, funduscopic examination revealed macular yellowish well-defined dots in 6 eyes of 3 patients and macular RPE dystrophy, with well-defined hypo-pigmented RPE areas, in both eyes of 1 patient. Two CHM carriers belonged to pattern A (**Figure 3**), 1 patient to pattern B, 2 patients to pattern C, and 1 patient to pattern D; therefore 4 patients (4/6) were characterized by RPE abnormalities both at the periphery and posterior pole (**Figure 2**).

**Table 1:** Clinical and genetic findings.

Clinical and OCT data			
	CHM carriers (n = 6)	controls (n = 7)	P value
Age, years mean (SE)	45.5 (5.92)	45.6 (5.50)	0.993
SphEq, diopters mean (SE)	0.08 (0.13)	−0.71 (0.51)	0.161
SFCT, μm mean (SE)	270.9 (17.53)	281.4 (16.23)	0.660
LA, mm^2^ mean (SE)	0.98 (0.07)	1.09 (0.06)	0.272
SA, mm^2^ mean (SE)	0.54 (0.03)	0.590 (0.02)	0.250
TCA, mm^2^ mean (SE)	1.53 (0.09)	1.68 (0.09)	0.245
CVI mean (SE)	63.9 (1.09)	65.2 (1.01)	0.400
CHM-carrier mutations			
CHM carriers	CHM mutations		
Patient P1	c.666_669del (p.Glu223Alafs*8)		
Patient P2	c.757C>T (p.Arg253*)		
Patient P3	(c.-29-?_1962+?del)		
Patient P4	(c.-29-?_1962+?del)		
Patient P5	c.666_669del (p.Glu223Alafs*8)		
Patient P6	c.1584_1587del (p. Val529Hisfs*7)		

SphEq, mean spherical equivalent; LA, luminal area; SA, stromal area; TCA, total choroidal area; CVI, choroidal vascularity index; SE, standard error.

### Choroidal Thickness Assessment and CVI Calculation

The mean SFCT was 270.9± 54.3 μm in CHM carriers and 281.4 ± 36.8 μm in controls (p = 0.660). The LA resulted 0.99 ± 0.25 mm^2^ in the CHM carrier group and 1.01 ± 0.13 mm^2^ in the controls (p = 0.272). The SA was 0.53 ± 0.09 mm^2^ for CHM carriers and 0.59 ± 0.07 mm^2^ for controls (p = 0.250) and TCA 1.53 ± 0.34 mm^2^ and 1.69 ± 0.19 mm^2^ respectively (p = 0.245). The mean CVI measured 64.03 ± 3.98% for the CHM carriers and 65.25 ± 2.55% for the controls (p = 0.400) (**Table 1**). Furthermore, we studied the subfoveal choroidal area in CHM carriers with (4/6) and without RPE impairment (2/6) within a 3 mm diameter centered over the fovea; the LA was 0.504 ± 0.10 mm^2^ and 0.587 ± 0.06 mm^2^, the SA was 0.264 ± 0.07 mm^2^ and 0.225 ± 0.05 mm^2^, the TCA was 0.742 ± 0.14 mm^2^ and 0.812± 0.10 mm^2^, and the CVI was 66.39 ± 4.27 and 72.44 ± 4.64 for CHM carriers with and without central RPE impairment, respectively. There were no significant differences (p > 0.05).

## Discussion

This study analyses the choroidal changes in CHM carriers using a biomarker recently introduced: the Choroidal Vascularity Index (CVI) (6, 8, 9). To our knowledge this is the first study to examine parameters such as the SA, LA, TCA, and CVI in CHM carriers and to compare the parameters with age- and sex-matched controls.

More specifically, we know that CHM carriers present fundus abnormalities early in the course of the disease (7, 10, 11), however we studied novel choroidal OCT parameters in order to quantify the choroidal abnormalities associated with retinal alterations; furthermore, these data could be helpful to better understand the pathogenic mechanism of choroideremia, which is still not clear. Different hypotheses have been suggested in literature with controversial theories: certain studies have proposed that the RPE degenerates first, followed by alteration of the choroid and photoreceptor (PHR) layer (12, 13); others that the photoreceptors are involved earlier while the RPE and choriocapillaris later (14, 15) or that independent damage of the RPE and PHRs occurs and is followed by deterioration of the choroid (16, 17); moreover, the histopathologic detection of gliosis led to the assumption that inflammation may play a role in the disease process (18).

Histological analyses have detected depigmentation and irregular thickness of the RPE, a thickened Bruch’s membrane, and a thinner hypocellular choroid in CHM carriers (12, 19). The results of our study do not show differences between carriers and controls regarding the SFCT. This finding is in accordance with previous studies that measured the SFCT using OCT (3, 7, 20), although it is known that this value tends to decrease with age (21, 22).

More recently, many authors did not observe retinal or choroidal vascular abnormalities in CHM female carriers using OCT-A (7, 23); more specifically, in a previous study, our group did not find any OCT-A differences between CHM carriers and controls when studying CHM female carriers who presented peripheral but also RPE alterations in the macular area (7). On the contrary, in young CHM patients, OCT-A revealed a significant impairment of vessel density in the retinal and choriocapillaris layers (7). These results supported a primary role of the RPE in CHM pathogenesis, followed by alteration in the choriocapillaris and, finally, in the retinal vasculature (7, 24).

Regarding the CVI in CHM, our group studied choroidal features in young and affected male patients (25). While a reduced CVI has been reported for other inherited retinal diseases, such as Stargardt disease, cone-rod dystrophy, retinitis pigmentosa, Best disease, and Bietti crystalline dystrophy (4–6), in young CHM male patients an unaltered CVI ratio was detected although the TCA, LA, and SA were reduced (25); the findings suggested a simultaneous impairment of the stromal and vascular components, which started from the peripheral areas (25). The reduced TCA and LA (caused by impairment of large and medium calibre choroidal vessels) were hypothesized to be secondary to choriocapillaris alterations (25).

In this study we did not observe differences in the CVI between CHM female carriers and controls; more specifically we did not detect differences in the LA, SA, and TCA either; these results are extremely important if we consider that in the majority of patients (4/6), we detected central RPE abnormalities. These results are in agreement with previous studies which did not find any choroidal abnormalities in CHM carriers compared to controls using both structural OCT and OCT-A (7, 23). Furthermore, Suzuki et al. (26) observed a normal SFCT and cone photoreceptor densities in CHM female carriers despite the presence of distinctive depigmentation of the RPE. We know that there is no general consensus regarding the correlation between CC impairment and CVI: although a reciprocal influence between CVI parameters and CC alteration is theoretically possible, a strong correlation has not been demonstrated since the CVI parameters may not be sensitive to small alterations of the CC layer (24, 25, 27,) for this reason, and according to the results of this study, we did not detect any impairment of the medium and large choroidal vessels in CHM carriers despite the presence of fundus abnormalities.

These findings further support the thesis already proposed regarding CHM pathogenesis (24 25): we believe that medium and large choroidal vessel abnormalities are not involved in CHM pathogenesis in carriers; the RPE is the first structure to undergo alterations in CHM carriers. Indeed, parameters such as the CVI, LA, SA, and TCA do not alter in CHM carriers who show RPE impairment at funduscopic examination, FAF and OCT (3).

The major limit of this study is the relatively low number of patients, although CHM is a rare disease. In particular, we have considered a specific population: CHM carriers, selected after an accurate study of all the family members, who were genetically characterized. We cannot exclude that some CHM carriers, characterized by a severe fundus phenotype (20) and therefore similar to CHM patients, could present an impairment of the LA, SA, and TCA.

## Conclusion

In conclusion, female CHM carriers were characterized by an LA, SA, TCA, and CVI similar to the controls. The finding suggests the presence of preserved choroidal vasculature in eyes with RPE impairment and, therefore, supports the primary role of the RPE, rather than alteration of the medium and large vessels of the choroid, in the pathogenesis of CHM disease in carriers.

## Data Availability Statement

The original contributions presented in the study are included in the article/supplementary files. Further inquiries can be directed to the corresponding author.

## Ethics Statement

The studies involving human participants were reviewed and approved by Careggi University Hospital Ethic Committe. The patients/participants provided their written informed consent to participate in this study.

## Author Contributions

All authors listed have made a substantial, direct, and intellectual contribution to the work and approved it for publication.

## Conflict of Interest

The authors declare that the research was conducted in the absence of any commercial or financial relationships that could be construed as a potential conflict of interest.

## Publisher’s Note

All claims expressed in this article are solely those of the authors and do not necessarily represent those of their affiliated organizations, or those of the publisher, the editors and the reviewers. Any product that may be evaluated in this article, or claim that may be made by its manufacturer, is not guaranteed or endorsed by the publisher.
